# Associations between the food environment and food and drink purchasing using large-scale commercial purchasing data: a cross-sectional study

**DOI:** 10.1186/s12889-022-14537-3

**Published:** 2023-01-10

**Authors:** Alexandra Kalbus, Laura Cornelsen, Andrea Ballatore, Steven Cummins

**Affiliations:** 1 Department of Public Health, Environments and Society, London School of Hygiene & Tropical Medicine, London, UK; 2 Department of Digital Humanities, King’s College London, London, UK

**Keywords:** Food, Food purchasing, Ultra-processed foods, Fruits and vegetables, Food environment, Neighbourhood, Food geography

## Abstract

**Background:**

Evidence for an association between the local food environment, diet and diet-related disease is mixed, particularly in the UK. One reason may be the use of more distal outcomes such as weight status and cardiovascular disease, rather than more proximal outcomes such as food purchasing. This study explores associations between food environment exposures and food and drink purchasing for at-home and out-of-home (OOH) consumption.

**Methods:**

We used item-level food and drink purchase data for London and the North of England, UK, drawn from the 2019 Kantar Fast Moving Consumer Goods panel to assess associations between food environment exposures and household-level take-home grocery (*n*=2,118) and individual-level out-of-home (*n*=447) food and drink purchasing. Density, proximity and relative composition measures were created for both supermarkets and OOH outlets (restaurants and takeaways) using a 1 km network buffer around the population-weighted centroid of households’ home postcode districts. Associations between food environment exposure measures and frequency of take-home food and drink purchasing, total take-home calories, calories from fruits and vegetables, high fat, salt and sugar products, and ultra-processed foods (UPF), volume of take-home alcoholic beverages, and frequency of OOH purchasing were modelled using negative binomial regression adjusted for area deprivation, population density, and individual and household socio-economic characteristics.

**Results:**

There was some evidence for an inverse association between distance to OOH food outlets and calories purchased from ultra-processed foods (UPF), with a 500 m increase in distance to the nearest OOH outlet associated with a 1.1% reduction in calories from UPF (IR=0.989, 95%CI 0.982–0.997, *p*=0.040). There was some evidence for region-specific effects relating to purchased volumes of alcohol. However, there was no evidence for an overall association between food environment exposures and take-home and OOH food and drink purchasing.

**Conclusions:**

Despite some evidence for exposure to OOH outlets and UPF purchases, this study finds limited evidence for the impact of the food environment on household food and drink purchasing. Nonetheless, region-specific effects regarding alcohol purchasing indicate the importance of geographical context for research and policy.

**Supplementary Information:**

The online version contains supplementary material available at 10.1186/s12889-022-14537-3.

## Introduction

Dietary risk factors have been linked to a variety of adverse health outcomes, including diabetes, cancer, and overweight and obesity [1]. Equally, excess alcohol consumption is associated with chronic disease, premature death and disability [2]. Energy-dense and nutrient-deficient, as well as ultra-processed foods have also been shown to be disadvantageous to health. Ultra-processed foods are linked to a higher energy intake and subsequently, obesity and other non-communicable diseases [3]. Foods consumed away from home are higher in energy, have greater salt and fat content, and are more processed than food prepared at home [4]. For instance, the majority of meals served in large UK restaurant and fast-food chains exceed the recommended energy content of a main meal [5, 6]. Currently, 28% of adults in England are obese and a further 36% are overweight [7]. Overweight and obesity as well as their related social inequalities are predicted to increase further over the next decade [8].

Environmental factors are associated with dietary behaviours in various ways. The retail food environment, often referred to as the ‘food environment’, constitutes the totality of physical food outlets available for consumers such as supermarkets, corner stores, restaurants, and takeaway outlets in a given geographical setting [9]. The main mechanism by which the food environment influences individual dietary behaviour is through differences in availability of, and access to, components of healthy and less healthy diets [10]. Availability and accessibility, commonly quantified as density and distance, are commonly referred to as absolute food environment exposure measures [11]. Other potential mechanisms are environmental cues prompting behavioural responses, and the implicit shaping of consumers’ norms on food choice through the composition of food environments, i.e. the relative density of outlets such as supermarkets, restaurants and takeaway outlets [12].

Although many previous studies have found associations between the food environment and dietary health outcomes, including diet, body weight and obesity [13], evidence mostly originates from the US. In the UK, evidence on the relationship between the food environment and individual outcomes is inconclusive [14]. While an analysis of data from the Fenland Study showed that greater exposure to fast food outlets was associated with fast food consumption and body weight [15], other studies have not replicated these findings [16, 17]. A potential reason for this discrepancy is the wide range of methods used to define and measure the food environment and relevant health and behavioural outcomes [18, 19]. A focus on more distal health outcomes such as overweight and obesity rather than the intermediate behavioural steps on the causal chain between food environment exposure and individual health outcomes may obscure the precise nature of any causal relationship. Even when considering more proximal outcomes such as food and drink purchasing and total diet, the quality of outcome data is often a limiting factor. Common methods such as diet recall surveys and food frequency questionnaires are well-known to be susceptible to bias [20]. Furthermore, studies often lack granularity, when food intake data are limited to a narrow, pre-defined set of food categories and/or a short period of time [19].

In the present study, we address these shortcomings by utilising large-scale objective consumer purchase data. We analyse the relationship between the food environment and food and drink purchasing in England, using absolute and relative exposure measures and a variety of food and drink purchasing measures. We also examine if these relationships differ by region.

## Methods

We use socio-demographic and objectively recorded consumer panel purchase data from 2,118 households. This includes item-level data on 3,413,588 purchased packs of take-home and 108,830 purchased packs of out-of-home (OOH) food and drink products collected over a 12-month period. Recorded food and drink purchases constitute objective measures which have been shown to reasonably reflect diet, while being less prone to bias [21].

### Food and drink purchasing data

Data on household food and drink purchasing for in-home and OOH consumption for 2019 were obtained from the Kantar Fast Moving Consumer Goods panel (FMCG) [22]. This is a live household consumer panel where purchases brought into the home are recorded with hand-held barcode scanners. Bespoke barcodes are provided for non-barcoded products such as loose fruits and vegetables. Kantar collects data on the nutritional content of products twice a year as well as uses product images provided by third-party supplier Brandbank. Where information cannot be obtained directly, nutritional values are either copied across from similar products, or an average value for the category or product type is calculated and used instead. Within this panel, a subsample of individuals reports OOH food and drink purchases through a mobile phone application. However, nutritional information for OOH products is unknown unless these are purchased from supermarkets. Data for this study comprised the regions Greater London and the North of England (North East, North West, and Yorkshire and the Humber) and were available from The TfL Study (study protocol: http://www.isrctn.com/ISRCTN19928803).

### Food and drink purchasing outcomes

Individual item transaction-level purchase data were aggregated to household-week level and averaged over 2019. Kantar data are routinely analysed aggregated to the weekly level [23, 24]. We created a range of purchasing outcome measures which capture food shopping behaviour, such as the frequency of food shopping and total calories, as well as those assessing the acquisition of foods favourable to health such as fruit and vegetables and those less favourable to health such as foods high in fat, salt and sugar, ultra-processed foods, and alcohol. Frequency of purchasing was defined as number of days per week with purchase occasions. Total energy purchased was defined as the average weekly calories (kcal) purchased per household member. Calories that households purchased from fruits and vegetables, foods and drinks high in fat, salt and sugar (HFSS), and ultra-processed foods (UPF) were expressed as a proportion of total calories purchased. Although overlap is likely, we included both HFSS and UPF classifications in the analysis, with the former emphasising the macronutrient composition and the latter the level of processing. While categorising foods and drinks as HFSS constitutes a policy-relevant classification in the UK, consumption using this categorisation has not been consistently associated with dietary health [25]. Consumption of UPF on the other hand has been linked to adverse health outcomes, but this classification is yet to be used in policies [3]. Fruits and vegetables were defined using a previously developed classification [26]. Products were classified as HFSS according to the Nutrient Profiling Model [27] as previously described [23]. UPF were determined following the NOVA classification [28] which was applied using Kantar’s proprietary product classifications. In some cases, product categories such as yoghurt were further differentiated to distinguish plain, ‘processed’ yoghurts from flavoured ‘ultra-processed’ products. Alcohol purchases were measured as the weekly volume (litres) of alcoholic beverages per adult household member. Food and drink purchasing outcomes described above refer to take-home purchases only, as nutritional information was not available for OOH purchasing. The frequency of OOH purchasing was calculated as the number of days with purchasing per 28-day sales period, referred to here as ‘month’.

### Food environment data

Postcode district of residence was the smallest geography available with which to assign a food environment exposure to each household. Postcodes are a geography primarily used by Royal Mail, the main UK postal service, to determine delivery areas [29]. Postcode districts are the first half of a postcode, for example, ‘NW5’, and vary in size. In our study sample, households were distributed over 621 postcode districts with a median size of 14.26 km^2^ (interquartile range 6.47, 36.24) and population of 32,960 (IQR 22,860, 42,795). We assigned each household to a location by using the population-weighted centroid of the postcode district. In doing so, we assumed that the most likely household location corresponds to the point closest to the majority of resident population within a postcode district. Neighbourhoods were defined as 1 km street network buffers around the centroid and were generated using ArcGIS Online. This 1 km buffer corresponds to a 15-minute walk and constitutes a common scale of exposure in food environment research [30].

**Fig. 1:**
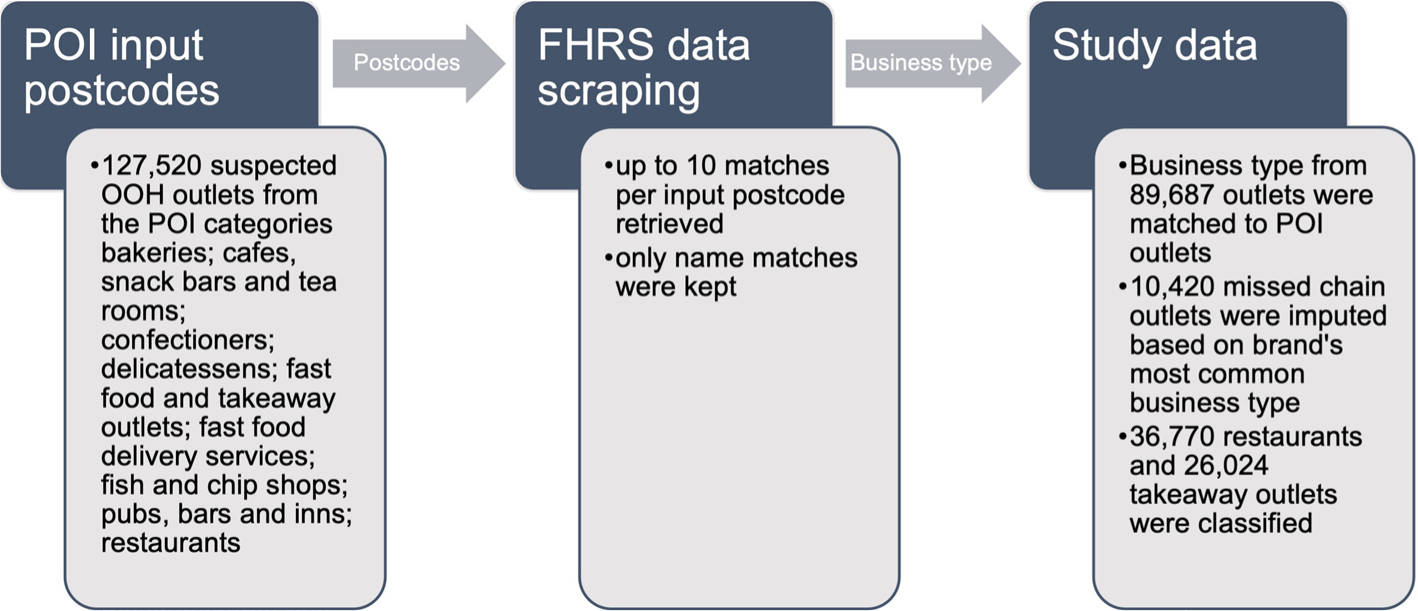
Cross-referencing process of POI food outlet data against the FHRS database. FHRS = Food Hygiene Rating Scheme, OOH = out of home, POI = Points of Interest. POI outlets were matched based on postcode and name to FHRS outlets

Data on food environment exposures were sourced from Ordnance Survey Points of Interest (POI) for March 2019 under an educational licence [31] and categorised into supermarkets, which included supermarkets and convenience stores, and OOH outlets, including takeaway food outlets and restaurants. Supermarkets were classified using a name-based approach according to Table 1. OOH outlets were categorised into ‘restaurants’ and ‘takeaways’ by cross-referencing POI data against the Food Hygiene Rating Scheme (FHRS) database published by the Food Standards Agency (FSA) [32], as shown in Fig. 1. The ‘business type’ recorded in the FHRS database corresponds to the use class of an outlet, a definition used when developing and implementing retail planning policy [33].

**Table 1:** Classification of supermarkets

Classification	Outlet description
Chain supermarkets	Supermarket chains (e.g. Tesco, Morrisons, Waitrose) and convenience symbol groups (e.g. Nisa, Co-op, Costcutter)
Independent supermarkets	Food retailers comprising of less than 5 outlets in POI data
All supermarkets	Chain supermarkets and independent supermarkets
excluded	Outlets selling primarily non-food items (e.g. newsstands) and outlets located in service stations

### Food environment exposures

Three types of food environment exposures were created: distance, density and composition measures. They were chosen to represent absolute measures of proximity and availability, and a relative measure of food environment composition [34]. For both supermarkets and OOH outlets, the distance from the inferred household address to the nearest outlet along the road network was determined using ArcMap version 10.5. Density of food outlets was calculated by dividing the count of respective outlets in the neighbourhood by its area (km^2^). Finally, the composition measure was built by comparing densities of OOH outlets and supermarkets in a neighbourhood. Accordingly, each neighbourhood was classified as having more supermarkets, more OOH outlets, or no outlets.

### Covariates

Included household sociodemographic characteristics were age (in years), sex, and social grade of the main food shopper, as well as number of adults and children (under 16 years) in the household. Social grade is a measure of occupational social status defined by the National Readership Survey (NRS), and includes the categories AB “Higher and intermediate managerial, administrative and professional”; C1 “Supervisory, clerical and junior managerial, administrative and professional”, C2 “Skilled manual workers”, D “Semi-skilled and unskilled manual workers”, and E “State pensioners, casual and lowest grade workers, unemployed with state benefits only”. Information was also available on the region and postcode district of residence for each household.

Population estimates for 2019 were retrieved from the Office for National Statistics [35] and interpolated from the lower layer super output area (LSOA) to the postcode district level. Population density in the postcode district was calculated by dividing the population by the postcode district’s area (km^2^). Area deprivation was approximated through the income deprivation domain of the Index of Multiple Deprivation England [36]. Income scores were interpolated from the LSOA to postcode district level. Then, postcode districts were internally ranked according to their income deprivation score.

### Analytical sample

We removed periods of two or more consecutive weeks of non-reporting from the take-home purchase data to address potential under-reporting, in line with previous reported work [37]. For OOH purchases, weeks were removed if they coincided with the household’s periods of underreporting take-home purchases. OOH purchases recorded by a household member other than the main OOH reporter were excluded.

### Statistical analysis

All statistical analyses and data management tasks, if not otherwise specified, were conducted with R version 4.0.5. Alpha was determined at 0.05.

Descriptive statistics and bivariate associations between purchase outcomes and food environment exposure were explored. To test for spatial dependency, we calculated Global Moran’s I using GeoDa software (see Additional file 1: Table S1). No spatial autocorrelation was detected, and we proceeded the multivariable analysis without accounting for spatial structure. Corresponding to the outcomes being over-dispersed count data, negative binomial regression models were chosen. Fixed- and random-effect models nested in postcode district as well as zero-truncated models and explicitly modelling zero-inflation were explored. Final model choice was guided by the Bayesian Information Criterion and Root Mean Square Error. Accordingly, all outcomes were modelled with fixed-effects negative binomial models which best fitted the data.^1^

Outcome measures were expressed as rates: Take-home purchase occasions per week; calories purchased per week and household size; calories from fruits and vegetables, HFSS, and UPF per total calories; volume of alcohol per week and adult household members; frequency of OOH purchasing per month. To account for these rates in negative binomial models, respective offsets, i.e. log terms with a coefficient of 1, were modelled.

Covariates adjusted for in all models comprised age, gender and social grade of the main shopper, number of adults and number of children in the household, region, area deprivation, and population density. Furthermore, interactions between region and social grade of the main shopper, area deprivation and population density were modelled to reflect the diversity between the two regions. Each of the seven exposures, shown in Table 2, was modelled separately. For take-home purchasing outcomes, we modelled aggregated OOH outlet exposure, and vice versa, we used aggregated supermarket exposure when modelling OOH purchasing. Distance measures were scaled to a 500 m difference to facilitate interpretation of coefficients.

**Table 2:** Food environment exposures examined in models for take-home and out-of-home purchasing

Take-home purchasing models	Out-of-home purchasing models
Density of chain supermarkets (count/km^2^)	Density of all supermarkets (count/km^2^)
Distance to nearest chain supermarket (m)	Distance to nearest supermarket (any) (m)
Density of independent supermarkets (count/km^2^)	Density of restaurants (count/km^2^)
Distance to nearest independent supermarket (m)	Distance to nearest restaurant (m)
Density of OOH outlets (count/km^2^)	Density of takeaway outlets (count/km^2^)
Distance to nearest OOH outlet (m)	Distance to nearest takeaway outlet (m)
Composition of the food environment - More supermarkets - More OOH outlets - No outlets	Composition of the food environment - More supermarkets - More OOH outlets - No outlets

Region-specific associations between food environment exposures and purchasing were examined by modelling an additional interaction term between region and the respective food environment exposure.

Multiple testing was addressed by adjusting *p* values following the Benjamini-Hochberg approach [38]. This is a method to control the false-discovery rate, i.e. the expected proportion of rejecting the null hypothesis when in fact it was true (type I error) and involves adjusting *p* values according to their rank within the set of tests. Subsequently, from the first null hypothesis to be rejected after adjustment of *p* values, all following hypotheses will be rejected, too. Compared to methods controlling the family-wise error rate such as the Bonferroni correction the Benjamini-Hochberg method has higher power [38].

### Sensitivity analysis

We examined robustness of observed results with respect to the choice of buffer for the density measures, the aggregation of supermarkets, and the inclusion of OOH purchases from a household member other than the main reporter. To assess if the chosen neighbourhood delineation of 1 km affects results, buffers of 0.5 km, 2 km and 5 km were explored. We assessed aggregations of big chain supermarkets, small chain supermarkets and convenience symbol groups, and independent supermarkets other than ‘chain supermarkets’ and ‘all supermarkets’. Finally, all OOH purchases, including those not reported from the main shopper for whom sociodemographic characteristics were not known, were examined.

## Results

The 2,118 households reporting take-home purchases and 447 individuals reporting OOH purchases were evenly distributed across London and the North of England. Table 3 and Table 4 display descriptive statistics for the take-home and OOH sample overall, and stratified by region.

**Table 3:** Description of the take-home sample

	Full sample (*N* = 2118)	London (*N* = 1063)	North of England (*N* = 1055)
Age of main shopper	53 (41, 62)	52 (42, 61)	53 (40, 63)
Gender of main shopper			
Female	1,537 (72.57%)	760 (71.50%)	777 (73.65%)
Male	581 (27.43%)	303 (28.50%)	278 (26.35%)
NRS social grade of main shopper			
AB	498 (23.51%)	287 (27.00%)	211 (20.00%)
C1	907 (42.82%)	476 (44.78%)	431 (40.85%)
C2	331 (15.63%)	133 (12.51%)	198 (18.77%)
D	234 (11.05%)	94 (8.84%)	140 (13.27%)
E	148 (6.99%)	73 (6.87%)	75 (7.11%)
Number of people in the household			
1	431 (20.35%)	259 (24.37%)	172 (16.30%)
2	765 (36.12%)	337 (31.70%)	428 (40.57%)
3	396 (18.70%)	186 (17.50%)	210 (19.91%)
4	383 (18.08%)	198 (18.63%)	185 (17.54%)
5+	143 (6.75%)	83 (7.81%)	60 (5.69%)
Children in the household			
Yes	617 (29.13%)	303 (28.50%)	314 (29.76%)
No	1,501 (70.87%)	760 (71.50%)	741 (70.24%)
Population density (people/km^2^)	3,426.65 (1,405.84, 5,776.85)	5,425.54 (4,121.96, 7,994.89)	1,462.75 (618.09, 2,702.78)
Area deprivation ^a^	-	338 (226, 471)	279 (116, 442)
Density of chain supermarkets (outlets/km^2^)	2.69 (1.25, 3.90)	3.11 (1.70, 4.50)	1.95 (0.62, 3.43)
Density of independent supermarkets (outlets/km^2^)	2.03 (0.65, 5.90)	4.92 (1.84, 10.38)	0.94 (0.00, 2.04)
Distance to nearest chain supermarket (m)	536.76 (321.79, 893.13)	403.06 (268.32, 724.45)	634.08 (428.64, 1,105.87)
Distance to nearest independent supermarket (m)	638.83 (323.42, 1,075.47)	419.15 (227.04, 691.68)	878.49 (581.90, 1,431.09)
Density of OOH outlets (outlets/km^2^)	7.91 (2.77, 18.35)	14.61 (6.00, 25.84)	4.55 (1.04, 9.32)
Distance to nearest OOH outlet (m)	486.39 (260.11, 778.69)	367.81 (199.64, 608.11)	615.55 (374.53, 969.87)
Neighbourhood food environment composition			
More supermarkets	496 (23.42%)	196 (18.44%)	300 (28.44%)
More OOH outlets	1,413 (66.71%)	826 (77.70%)	587 (55.64%)
No outlets	209 (9.87%)	41 (3.86%)	168 (15.92%)
Purchase occasions (days/week)	1.65 (1.10, 2.44)	1.73 (1.12, 2.52)	1.54 (1.10, 2.37)
Total kcal (kcal) ^b^	10,300.70 (7,349.43, 13,927.95)	9,769.06 (7,073.43, 13,125.83)	10,801.87 (7,696.76, 14,479.79)
kcal from fruit & vegetables (%)	3.98 (2.60, 5.86)	4.36 (2.86, 6.56)	3.68 (2.42, 5.22)
kcal from HFSS foods (%)	52.97 (47.05, 58.73)	52.47 (46.22, 58.37)	53.45 (48.06, 58.97)
kcal from UPF (%)	58.88 (49.73, 67.54)	56.94 (46.71, 66.14)	61.28 (52.49, 68.75)
Volume of alcohol (l) ^c^	0.15 (0.02, 0.50)	0.10 (0.02, 0.34)	0.22 (0.04, 0.64)

Values are percentages for categorical variables and median (interquartile range) for continuous variables. OOH outlets = outlets for out-of-home consumption, include restaurants and hot food takeaways; *HFSS* high in fat, salt and sugar (according to the Nutrient Profiling Model (UK Department of Health, 2011)); *UPF* ultra-processed foods (according to the NOVA classification (Monteiro et al., 2019))

^a^Median rank of income deprivation (ranks from 1 to 630). The lower the rank, the more deprived is the area

^b^per household member and week

^c^per adult and week

**Table 4:** Description of the out-of-home sample

	Full sample (*N* = 447)	London (*N* = 204)	North of England (*N* = 243)
Age of main shopper	51 (42, 60)	51 (42, 59)	51 (40, 60)
Gender of main shopper			
Female	324 (72.48%)	145 (71.08%)	179 (73.66%)
Male	123 (27.52%)	59 (28.92%)	64 (26.34%)
Social grade of main shopper			
AB	107 (23.94%)	56 (27.45%)	51 (20.99%)
C1	210 (46.98%)	96 (47.06%)	114 (46.91%)
C2	67 (14.99%)	28 (13.73%)	39 (16.05%)
D	45 (10.07%)	17 (8.33%)	28 (11.52%)
E	18 (4.03%)	7 (3.43%)	11 (4.53%)
Number of people in the household			
1	99 (22.15%)	60 (29.41%)	39 (16.05%)
2	165 (36.91%)	63 (30.88%)	102 (41.98%)
3	81 (18.12%)	34 (16.67%)	47 (19.34%)
4	79 (17.67%)	35 (17.16%)	44 (18.11%)
5+	23 (5.15%)	12 (5.88%)	11 (4.53%)
Children in the household			
Yes	132 (29.53%)	53 (25.98%)	79 (32.51%)
No	315 (70.47%)	151 (74.02%)	164 (67.49%)
Population density (people/km^2^)	3464.79 (1392.62, 5622.98)	5604.90 (4283.04, 8030.15)	1517.30 (662.04, 3117.36)
Area deprivation ^a^	-	163 (110, 227)	136 (53, 227)
Density of supermarkets (outlets/km^2^)	5.39 (2.27, 10.12)	9.25 (4.37, 17.00)	3.46 (1.23, 6.19)
Distance to nearest supermarkets (m)	463.31 (251.41, 724.45)	298.03 (160.77, 526.03)	596.34 (373.68, 890.86)
Density of restaurants (outlets/km^2^)	3.47 (0.75, 11.46)	9.54 (4.19, 19.64)	1.44 (0.00, 3.88)
Distance to nearest restaurant (m)	572.05 (334.20, 1,012.09)	370.54 (202.33, 619.87)	788.80 (497.46, 1,394.71)
Density of takeaway outlets (outlets/km^2^)	4.32 (1.39, 8.49)	5.74 (2.73, 10.27)	3.35 (0.78, 6.58)
Distance to nearest takeaway outlet (m)	518.08 (289.35, 879.82)	420.56 (214.11, 643.81)	633.01 (398.33, 1,066.59)
Neighbourhood food environment composition			
More supermarkets	92 (20.58%)	33 (16.18%)	59 (24.28%)
More OOH outlets	307 (68.68%)	165 (80.88%)	142 (58.44%)
No outlets	48 (10.74%)	6 (2.94%)	42 (17.28%)
OOH purchase occasions (days/month)	4.15 (2.27, 7.63)	4.25 (2.29, 7.77)	4.00 (2.20, 7.23)

*OOH* out-of-home. Values are percentages for categorical variables and median (interquartile range) for continuous variables

^a^Median rank of income deprivation (ranks from 1 to 298). The lower the rank, the more deprived is the area

Household exposure to OOH outlets was greater than for supermarkets, with two thirds of neighbourhoods having more OOH outlets than supermarkets (66.7% and 68.7% in take-home and OOH sample, respectively). No food outlets were present in 9.9% of neighbourhoods in the take-home, and 10.7% in the OOH sample. Overall exposure to the food environment was greater in the OOH sample than in the take-home sample, and greater in London compared to the North of England, with disproportionally more OOH outlets and independent supermarkets.

Households purchased food and drinks for take-home consumption on median 1.7 days per week. Median purchased energy from foods and drinks brought to the home was 10,301 calories per household member per week. Of the purchased calories, 4% were from fruits and vegetables, 53% from HFSS, and 58.9% from UPF. The median weekly volume of purchased alcoholic beverages for at-home consumption was 0.15 litres per adult. Individuals reported OOH purchases on a median 4.2 days per month.

In London, more main household shoppers were in higher social grades, and households resided in less deprived and more densely populated areas than their counterparts in the North of England. London households purchased take-home food and beverages more frequently, sourced lower volumes of alcoholic beverages, fewer total calories, fewer calories from HFSS and UPF, and more calories from fruits and vegetables. Individuals in London also reported slightly more OOH purchase occasions per month.

Bivariate analysis showed that more deprived and more densely populated areas were associated with greater exposure to food outlets. Additional file 1: Tables S2–S5 contains the full bivariate analysis.

### Associations between food environment exposures and purchases

Although the bivariate analysis (see Additional file 1: Tables S2 and S3) suggested some evidence of a relationship between food environment exposure and food and drink purchasing outcomes, after controlling for covariates and adjusting for multiple testing (Table 5 and Table 6) there was no evidence for a consistent relationship. There was moderate evidence for a small association between the distance to the nearest OOH outlet and calories purchased from UPF. For each increase of 500 m in the distance to the nearest OOH outlet, take-home UPF calories decreased by 1.1% (Incidence rate=0.989, 95% confidence interval 0.982–0.997, *p*=0.040).

**Table 5:** Parameter estimates and 95% CI of take-home purchase outcomes associated with food environment exposures (main effects)

	Adjusted Estimates																	
	Frequency			Total Calories			Calories from fruit & vegetables			Calories from HFSS			Calories from UPF			Alcohol volume		
Exposure	IR	95% CI	*p* value	IR	95% CI	*p* value	IR	95% CI	*p* value	IR	95% CI	*p* value	IR	95% CI	*p* value	IR	95% CI	*p* value
Density of chain supermarkets	1.006	0.994; 1.017	0.436	1.006	0.997; 1.015	0.760	0.999	0.985; 1.013	0.903	1.003	0.999; 1.008	0.318	1.005	1.000; 1.011	0.147	0.965	0.928; 1.004	0.197
Distance to chain supermarkets	0.989	0.977; 1.002	0.251	1.002	0.992; 1.013	0.760	1.004	0.988; 1.020	0.903	0.998	0.993; 1.003	0.517	0.993	0.987; 1.000	0.147	1.004	0.960; 1.049	0.875
Density of independent supermarkets	0.998	0.993; 1.004	0.614	1.001	0.996; 1.005	0.760	1.002	0.994; 1.009	0.903	0.998	0.995; 1.000	0.261	0.998	0.995; 1.001	0.183	0.979	0.960; 0.998	0.189
Distance to independent supermarkets	0.989	0.979; 1.000	0.222	0.998	0.989; 1.007	0.760	1.001	0.988; 1.015	0.903	1.000	0.995; 1.004	0.832	0.996	0.991; 1.002	0.231	0.996	0.959; 1.034	0.875
Density of OOH outlets	1.001	0.999; 1.003	0.382	1.000	0.998; 1001	0.760	1.000	0.998; 1.002	0.903	1.000	0.999; 1.000	0.517	1.000	0.999; 1.001	0.648	0.995	0.990; 1.001	0.197
Distance to OOH outlets	0.990	0.976; 1.005	0.362	1.003	0.991; 1.015	0.760	1.009	0.991; 1.028	0.903	0.996	0.990; 1.001	0.318	0.989	0.982; 0.997	0.040	1.021	0.970; 1.073	0.686
Food environment composition																		
More OOH outlets	0.996	0.948; 1.047	0.879	0.978	0.939; 1.019	0.760	1.047	0.983; 1.115	0.607	0.984	0.965; 1.004	0.318	0.977	0.952; 1.002	0.147	1.057	0.888; 1.257	0.711
No outlets	0.917	0.847; 0. 991	0.222	0.976	0.915; 1.041	0.760	1.078	0.976; 1.190	0.607	0.981	0.951; 1.012	0.363	0.971	0.932; 1.010	0.194	1.317	1.003; 1.730	0.189

*95% CI* 95% confidence interval, *HFSS* high in fat, salt and sugar, *IR* Incidence Rate, *OOH* out of home, *UPF* ultra-processed foods. Effect estimates of density measures refer to a change in incidence rate in response to an increase of 1 m/km^2^. Effect estimates of distance measures refer to a change in incidence rate in response to an increase of 500 m. The reference category for the composition of food environments is neighbourhoods with more supermarkets

All models are adjusted for age, sex and NRS social grade of the main shopper, number of children and adults in the household, region, area deprivation and population density, and interactions between region and NRS social grade, area deprivation, and population density. *p* values were adjusted for multiple testing using the Benjamini-Hochberg method

**Table 6:** Parameter estimates and 95% CI of OOH purchasing associated with food environment exposures (main effects)

Exposure	IR	95% CI	*p* value
Density of all supermarkets	0.979	0.961; 0.998	0.079
Distance to any supermarket	1.012	0.931; 1.101	0.875
Density of restaurants	0.989	0.980; 0.998	0.079
Distance to restaurants	1.005	0.952; 1.060	0.875
Density of takeaway outlets	0.976	0.955; 0.997	0.079
Distance to takeaway outlets	1.004	0.951; 1.061	0.875
Composition of food environments			
More OOH	0.850	0.685; 1.056	0.283
No outlets	0.861	0.622; 1.191	0.584

*95% CI* 95% confidence interval, *OOH* out of home, *IR* Incidence Rate. Effect estimates of density measures refer to a change in incidence rate in response to an increase of 1 m/km^2^. Effect estimates of distance measures refer to a change in incidence rate in response to an increase of 500 m. The reference category for the composition of food environments is neighbourhoods with more supermarkets

All models are adjusted for age, sex, NRS social grade, number of children and adults in the household, region, area deprivation and population density, and interactions between region and NRS social grade, area deprivation, and population density. *p* values were adjusted for multiple testing using the Benjamini-Hochberg method

### Region-specific associations between food environment exposures and purchasing

Table 7 and Table 8 contain the results of the region-specific analysis. There was evidence of effect modification by region in the relationships between total take-home calories purchased and food environment composition (*p*=0.031); and take-home volume of alcohol purchased and the density of independent supermarkets (*p*=0.028) and distance to OOH outlets (*p*=0.028). Interaction terms are shown in Additional file 1: Tables S6 and S7. Despite effect modification by region for associations between food environment composition and purchased take-home calories, there were no statistically significant associations observed in either region. Region-specific associations were observed for purchased volume of take-home alcoholic beverage outcomes: there was strong evidence for an inverse relationship between density of independent supermarkets and purchased alcohol volume in the North of England (IR=0.952, 95%CI 0.927–0.978, *p*=0.003), but not in London. Furthermore, an increase of 500 m in the distance to the nearest OOH outlet was associated with a 13.9% increase in take-home purchased volume of alcohol in the North of England, and with a 29.8% increase in London (IR=1.139, 95%CI 1.039–1.248, *p*=0.023 and IR=1.298, 95%CI 1.089–1.549, *p*=0.030, respectively)

**Table 7:** Region-specific parameter estimates and 95% CI of take-home purchase outcomes associated with food environment exposures

		Adjusted Estimates																	
		Frequency			Total Calories			Calories from FV			Calories from HFSS			Calories from UPF			Alcohol volume		
Exposure	Region	IR	95% CI	*p* value	IR	95% CI	*p* value	IR	95% CI	*P* value	IR	95% CI	*p* value	IR	95% CI	*p* value	IR	95% CI	*p* value
Density of chain supermarkets	London	1.004	0.988; 1.021	0.900	1.010	0.997; 1.024	0.545	0.997	0.977; 1.018	0.891	0.999	0.992; 1.005	0.788	1.004	0.996; 1.013	0.693	0.963	0.910; 1.020	0.502
	NE	1.006	0.994; 1.017	0.646	1.006	0.997; 1.016	0.514	0.999	0.985; 1.013	0.988	1.003	0.998; 1.007	0.531	1.005	0.999; 1.1011	0.335	0.965	0.928; 1.004	0.150
Distance to chain supermarkets	London	1.033	0.992; 1.077	0.475	1.013	0.979; 1.048	0.724	1.018	0.967; 1.073	0.891	1.009	0.992; 1.025	0.415	1.004	0.983; 1.026	0.922	1.099	0.952; 1.268	0.502
	NE	1.009	0.987; 1.031	0.646	1.007	0.989; 1.025	0.514	1.010	0.983; 1.038	0.763	1.003	0.994; 1.011	0.807	0.998	0.987; 1.009	0.741	1.046	0.970; 1.128	0.322
Density of independent supermarkets	London	0.998	0.992; 1.004	0.900	1.000	0.995; 1.005	0.981	1.002	0.994; 1.009	0.891	0.997	0.994; 0.999	0.067	0.998	0.995; 1.001	0.693	0.988*	0.967; 1.009	0.502
	NE	0.998	0.990; 1.006	0.704	1.002	0.996; 1.009	0.514	1.004	0.994; 1.013	0.763	0.999	0.996; 1.002	0.807	0.997	0.993; 1.001	0.363	0.952*	0.927; 0.978	0.003
Distance to independent supermarkets	London	0.995	0.954; 1.039	0.900	1.018	0.984; 1.054	0.724	0.998	0.946; 1.053	0.940	1.014	0.997; 1.031	0.176	1.018	0.996; 1.040	0.693	0.988	0.852; 1.146	0.875
	NE	0.992	0.971; 1.014	0.646	1.008	0.990; 1.026	0.514	1.000	0.972; 1.028	0.988	1.006	0.997; 1.015	0.531	1.006	0.995; 1.018	0.538	0.992	0.919; 1.071	0.844
Density of OOH outlets	London	1.001	0.999; 1.003	0.873	1.000	0.998; 1.002	0.981	1.001	0.998; 1.003	0.891	0.999	0.999; 1.000	0.176	1.000	0.999; 1.001	0.958	0.997	0.990; 1.004	0.609
	NE	1.001	0.999; 1.003	0.646	0.999	0.998; 1.001	0.514	1.000	0.998; 1.002	0.988	1.000	0.999; 1.001	0.807	1.000	0.999; 1.001	0.741	0.995	0.989; 1.001	0.150
Distance to OOH outlets	London	1.054	1.002; 1.109	0.336	1.019	0.977; 1.062	0.724	1.030	0.966; 1.099	0.891	1.001	0.981; 1.021	0.926	0.999	0.974; 1.026	0.958	1.298*	1.089; 1.549	0.030
	NE	1.018	0.992; 1.046	0.646	1.010	0.989; 1.032	0.514	1.019	0.985; 1.053	0.753	0.998	0.988; 1.009	0.807	0.994	0.981; 1.007	0.602	1.139*	1.039; 1.248	0.023
Composition of food environments																			
More OOH	London	0.995	0.992; 1.074	0.900	1.013	0.952; 1.078	0.919	1.078	0.978; 1.187	0.891	0.973	0.944; 1.003	0.176	0.981	0.944; 1.021	0.693	1.042	0.799; 1.360	0.869
	NE	0.997	0.948; 1.048	0.895	0.984	0.944; 1.025	0.514	1.051	0.987; 1.121	0.577	0.983	0.964; 1.003	0.531	0.978	0.953; 1.003	0.335	1.054	0.885; 1.257	0.633
No outlets	London	1.013	0.868; 1.182	0.900	1.142*	1.006; 1.296	0.315	1.121	0.922; 1.364	0.891	1.052	0.989; 1.119	0.176	1.030	0.951; 1.115	0.749	1.165	0.679; 1.998	0.772
	NE	0.948	0.867; 1.037	0.646	1.025*	0.952; 1.103	0.514	1.089	0.971; 1.220	0.577	1.006	0.970; 1.042	0.807	0.990	0.945; 1.036	0.741	1.264	0.924; 1.729	0.228

*95% CI* 95% confidence interval, *IR* Incidence Rate, *NE* North of England, *OOH* out of home

*Effect interaction was detected (*p*<0.005)

Effect estimates of density measures refer to a change in incidence rate in response to an increase of 1 m/km^2^. Effect estimates of distance measures refer to a change in incidence rate in response to an increase of 500 m. The reference category for the composition of food environments is neighbourhoods with more supermarkets

All models were adjusted for age, sex and NRS social grade of the main shopper, number of children and adults in the household, region, area deprivation and population density, and interactions between region and NRS social grade, area deprivation, and population density. *p* values were adjusted for multiple testing using the Benjamini-Hochberg method

**Table 8:** Region-specific parameter estimates and 95% CI of OOH purchasing associated with food environment exposures

	Adjusted Estimates			
Exposure	Region	IR	95% CI	*p* value
Density of all supermarkets	London	0.986	0.964; 1.008	0.569
	NE	0.976	0.956; 0.996	0.093
Distance to any supermarket	London	1.007	0.805; 1.260	0.953
	NE	1.010	0.895; 1.140	0.983
Density of restaurants	London	0.991	0.978; 1.005	0.569
	NE	0.989	0.980; 0.999	0.093
Distance to restaurants	London	1.079	0.876; 1.329	0.763
	NE	1.038	0.931; 1.156	0.707
Density of takeaway outlets	London	0.987	0.954; 1.021	0.763
	NE	0.978	0.957; 0.999	0.112
Distance to takeaway outlets	London	0.992	0.821; 1.200	0.953
	NE	0.999	0.905; 1.103	0.983
Composition of food environments				
More OOH	London	0.742	0.524; 1.052	0.569
	NE	0.830	0.664; 1.036	0.200
No outlets	London	0.857	0.401; 1.831	0.921
	NE	0.874	0.574; 1.331	0.707

*95% CI* 95% confidence interval, *OOH* out of home, *IR* Incidence Rate, *NE* North of England. Effect estimates of density measures refer to a change in incidence rate in response to an increase of 1 m/km^2^. Effect estimates of distance measures refer to a change in incidence rate in response to an increase of 500 m. The reference category for the composition of food environments is neighbourhoods with more supermarkets

All models were adjusted for age, sex NRS social grade, number of children and adults in the household, region, area deprivation and population density, and interactions between region and NRS social grade, area deprivation, and population density. *p* values were adjusted for multiple testing using the Benjamini-Hochberg method

Although no effect modification was detected, it is worth noting that in both regions separately, there was no evidence for an association between the distance to OOH outlets and take-home calories from UPF. No region-specific associations involving OOH purchasing frequency were observed.

### Sensitivity analysis

Sensitivity analyses (see Additional file 1: Tables S8–S11) revealed that results were sensitive to the choice of buffer size, with observed associations changing size and direction when choosing different buffer sizes, but they generally remained non-significant and were in no apparent relationship with the chosen buffer size. Observed associations were robust to the aggregation of supermarket definitions and the inclusion of all OOH purchases instead of only those from the main reporter.

## Discussion

### Summary of findings

This study aimed to explore associations between three types of food environment exposure and objective measures of food and drink purchasing in England. We did not observe any consistent patterns of association between food environment exposure and food and drink purchasing for both take-home and out-of-home purchases, and found limited evidence of region-specific associations. The only associations we found were between the distance to the nearest OOH outlet and take-home purchased calories of UPF, and region-specific associations between food environment exposure and purchased volume of take-home alcoholic beverages.

### Interpretation and implication of findings

Calories purchased from UPF in this study constituted almost 59% of total calories purchased, an increase from a previous estimate of 57% for 2008-14 [39]. To our knowledge, this is the first investigation linking food environment exposure and UPF purchases in the UK. We found evidence for a small association between proximity to the nearest OOH outlet and take-home calories purchased from UPF. One potential explanation is that local OOH outlets may act as environmental cues for the purchase of certain types of food and drink for take-home consumption, particularly for individuals who prefer to eat at home rather than away from home. The neighbourhood food environment may set normative ‘benchmarks’ of consumers’ choice [40], which may explain the link between OOH food outlets and purchasing for at-home consumption. However, this finding may also be biased due to exposure misclassification given that households’ precise address locations were unknown, resulting in inaccurate proximity measurement.

Previous work suggests some evidence for an association between outlets selling alcohol for consumption off the premises, but mostly points towards a more complex relationship [41]. Although no main effects were observed, there was evidence of effect modification by region on the relationship between the volume of take-home alcohol and the density of independent supermarkets and distance to the nearest OOH outlet. Density of independent supermarkets was negatively associated with purchased alcohol volume in the North of England. The distance to the nearest OOH outlet was positively associated with volume of alcoholic beverages in both regions, with a stronger association observed in London. These relationships could result from both bulk buying and less consumption of alcoholic beverages away from home in areas with less access to food outlets, and needs to be considered within the context of different magnitude of food environment exposure in the study regions. The current study did not examine the occurrence of pubs and bars in neighbourhoods, but if they co-locate with other food retailers, households in areas with lower food environment exposure may also have fewer options to drink away from home. We also did not examine off-licences within this study.

The region-specific associations observed for the purchased volume of take-home alcoholic beverages allude to the importance of geographical context when designing research studies as well as interventions. In terms of the studied regions, London is often regarded as very different from the rest of England with respect to its population structure and composition, culture, economy, and built environment. It seems reasonable to assume that among other area characteristics, the exposure to certain aspects of the food environment may have different meanings to individuals in different geographical contexts.

Apart from those reported above, no pattern of associations was found. This is consistent with the current equivocal evidence for the association between food environment and individual outcomes in the UK [16]. Shareck et al., for example, found no evidence for a relationship between absolute food environment exposure and fast-food and sugar-sweetened beverage intake, but some evidence for an association with relative exposure to convenience stores, underlining the relevance of exposure classification [10]. An analysis of the Yorkshire Health Study found no relationship between fruit and vegetable consumption and neither the density of shops selling fruits and vegetables and fast-food outlets, nor the diversity of the food environment [17]. In contrast, an analysis of the Fenland Study in Cambridgeshire found evidence for an association between greater fast-food exposure and greater fast-food consumption and body mass index [15]. This suggests that a universal pattern of association is unlikely, but there may be geographical heterogeneity in patterns of exposure-outcome associations that is affected by wider contextual factors. Work by Mason et al. indicates that this might be true using data from the UK Biobank [42]. This may explain why national studies produce less consistent evidence on the association between food environment and health and behavioural outcomes than studies focusing on one geographical setting.

The limited evidence on associations between the food environment and individual outcomes in the UK is generally based on small effect sizes in well-powered studies [43]. Hence, true associations may be small. This may appear in contrast to the US, where evidence more consistently supports greater effects [13]. But the different societal and environmental contexts need to be considered, specifically the retail structure in the UK, with most urban residents having reasonable access to food outlets [44]. In addition, many studies would be underpowered to detect small effects, adding to the inconclusive evidence base.

Another potential reason for the inconclusive evidence in food environment research in the UK is the inconsistency in methods, including definition of exposure and outcome measures, and temporal and spatial scales [18].

Our study took advantage of granular purchase outcome data from a large sample, making it less prone to bias. Food environment research often focuses on distal outcomes on the causal chain such as weight status. Considering that within the time between food environment exposure and manifestation of outcomes, the latter could have been influenced by many other individual or environmental factors, proximal outcomes such as diet or even food and drink purchases may be more appropriate. There are many studies focusing on diet and nutritional intakes which are primarily measured using food frequency questionnaires and dietary recalls, both subjective measures. Few food environment studies use food and drink purchasing as outcome, and while some use household receipts [45], most rely on participant self-reported data [19], and none use large-scale commercial food and drink purchase data.

Despite high quality-outcome data, potential misclassification of exposure is a key limitation of our study. Comprehensive purchase data at transaction level and accompanied by nutritional information facilitated highly granular outcome measures. In contrast, exposure measures were less accurate as data confidentiality allowed us to use postcode districts as our smallest unit of geographical aggregation. Using the population-weighted centroid of a postcode district as a proxy for a household’s address likely introduced spatial error into the exposure metrics [46]. Resulting misclassification of exposure has been shown to bias effect estimates towards the null, which could be the reason for the absence of evidence in the present study [47]. However, Healy and Gilliland also showed that spatial accuracy of area aggregation is better for urban than rural areas [46]. As the majority of households in our study live in urban postcode districts, this error might be reduced. Further, if we assume that the spatial error is randomly distributed across the sample, our results are internally valid.

Our work demonstrates the trade-off between accuracy in outcome and exposure data when utilising commercial data such as Kantar FMCG. Further research is needed to reduce spatial error when using large-scale consumer data. For the year 2015 and region Greater London, loyalty card purchase data are available at the LSOA level [48]. While still being a spatial aggregation that requires some assumptions as to the household location, this aggregation level is considerably smaller than the postcode district available in the Kantar FMCG data and allows for more meaningful association between the environment and individual. Future data protection agreements with commercial partners could explore options to make data available at smaller spatial aggregations such as the LSOA level. Future research examining granular purchase data and their relationship with people’s environment should: a) be more spatially explicit, ideally on the basis of panellists’ home addresses; b) consider food environments in addition the home food environment such as the workplace; c) assess in-store food environments [49]; and d) be context-specific by not only accounting for the geographical, but also individual context, by for example including individual mobility and available modes of transport [17] and/or controlling for individual interaction with the food environment [50].

Finally, as the analysed data predate the COVID-19 pandemic, it can be assumed that the relationship between the home food environment and food and drink purchasing might have changed during periods of implemented stay-at-home orders in the UK and longer-term shifts in consumer food purchasing behaviour due to greater working from home. With individuals spending more time at home, the immediate neighbourhood food retail system becomes more important [51]. As such, pandemic-induced exposure to the residential food environment might present a unique opportunity to investigate relationships between the immediate neighbourhood’s food environment and individual purchasing behaviour, with a reduction in the bias introduced by other food environments such as those at work and school.

### Strengths and limitations

This study has several strengths. Firstly, we used large-scale objectively recorded food and drink purchasing data collected using barcode scanners that included detailed nutritional information on individual purchased items. To our knowledge, this is the first investigation that links large-scale food and drink purchasing data to food environment exposure measures in the UK. Secondly, the large geographical scale including areas in London and the North England enabled the investigation of region-specific associations between the food environment and food and drink purchasing. Lastly, outcome measures captured various behavioural and health-related aspects of food and drink purchasing, including two measures that capture unfavourable dietary components (HFSS and UPF purchases).

Several limitations of our work need to be considered. Firstly, it is unknown if the home food environment as operationalised in this study is the relevant spatial scale of exposure. The modifiable areal unit problem suggests that observed effects may depend on the delineation of scale, i.e. the neighbourhood [52]. In our study, the choice of buffer size did not determine the presence of associations between density measures and food and drink purchase outcomes, although the size and direction of effects varied across different buffer sizes. This emphasises the relevance of theoretically-informed rather than data-driven neighbourhood delineations [53]. Even if the home food environment was specified correctly, it is unlikely to be the only relevant environment for individuals’ food choices. For example, there is some evidence that suggests cumulative exposure through school/work and home food environments may be more strongly associated with dietary outcomes than each independent exposure alone [10, 15]. By limiting our study to the exposure to physical food outlets, we did not account for the small but increasing availability of online grocery and takeaway delivery. However, we assume that online services did not account for a large proportion of foods and drinks bought for at-home and OOH consumption. Online groceries for example only contributed 9.92% of total transactions in our sample. Secondly, instead of individual household addresses, only the postcode district of each study household was available as a result of data protection agreements. By inferring addresses using population-weighted centroids, introduction of spatial error is possible [46]. Especially proximity measures may be biased through incorrect address specification. A simulation study has found that median distance discrepancies resulting from inferring addresses from larger spatial units can be as high as 343 m and 2088 m in urban and rural areas, respectively [46]. Thirdly, the OOH sample, as a subsample of the take-home sample, is about one fifth the size of the total sample. Hence, analyses have lower power to detect potential associations. However, a smaller sample can still be informative when assessing associations between food environment exposures and purchasing. Fourthly, POI and FSA food environment data may not fully capture all operating food outlets, though validation studies suggest both are highly accurate [54]. Fifthly, our category-based approach to classifying UPF may not have captured all respective foods in the dataset. Inconsistent classification across studies is a common limitation of the NOVA system, which as of now lacks standardised, context-specific classification guidelines, partly because lists of ingredients are not regularly recorded in purchase or consumption datasets [55]. Finally, applying the same parameter specification to model all outcomes may not result in optimal model fit for every outcome.

## Conclusions

In this paper we investigated the relationship between food environment exposures and food and drink purchasing in England, using large-scale data. We found evidence for an association between proximity to OOH outlets and take-home calories from UPF as well as for region-specific associations between food environment exposure and purchased take-home volume of alcoholic beverages. Apart from these findings, we did not find consistent patterns of relationships between food environment exposure and food and drink purchasing. Nonetheless, our findings indicate the relevance of wider geographical context. Researchers and policy makers should tailor efforts to the specific context, as relationships may differ from one region to another.

As the current investigation was restricted to the home food environment, further research should combine the objectivity and granularity of consumer purchase data with spatially explicit, context-specific food environment exposure data, while accounting for differences in individual contexts.

## Supplementary Information

**Additional file 1.**

## Data Availability

Data on household socio-demographics and food and drink purchases were purchased from Kantar and cannot be shared due to contractual agreements. All other data, including area characteristics, are publicly available and sources are cited accordingly: Food outlet data are available from Ordnance Survey (OS) Points of Interest (https://www.ordnancesurvey.co.uk/business-government/products/points-of-interest) and the Food Standards Agency (https://ratings.food.gov.uk/). Some restrictions apply to the OS data which were obtained under an educational licence for this study. Population estimates were retrieved from the Office for National Statistics (https://www.ons.gov.uk/peoplepopulationandcommunity/populationandmigration/populationestimates/datasets/lowersuperoutputareamidyearpopulationestimates). Income deprivation scores were obtained from the Department of Housing, Communities & Local Government (https://www.gov.uk/government/statistics/english-indices-of-deprivation-2019). The analytical dataset, excluding contractually restricted commercial data, is available from the corresponding author upon request.

## Footnotes

1 Calories from fruits and vegetables, HFSS and UPF could also be understood as proportions of the total calories. Hence, beta regression models with a distribution capped between 0 and 1 were explored. However, because of considerations of the validity of beta models in this context, specifically regarding the varying denominators among the different measures, negative binomial models were reported. Results were identical from both types of models.
