# Supplementary material for: Associations between the food environment and food and drink purchasing using large-scale commercial purchasing data: a cross-sectional study
Source: BMC Public Health. 2023 Jan 10;23:72. doi: 10.1186/s12889-022-14537-3 (PMC9831883; doi:10.1186/s12889-022-14537-3)
Supplement: Supplementary file 1 — Additional file 1. [file 12889_2022_14537_MOESM1_ESM.docx]

| **Table S1**. Global Moran’s I for purchase outcomes | | | |
| --- | --- | --- | --- |
| Outcome | Full sample | London | North of England |
| Frequency | 0.007 | 0.017 | -0.004 |
| Total calories | 0.034 | 0.037 | 0.012 |
| % calories from fruit & veg | 0.010 | 0.003 | 0.002 |
| % calories from HFSS foods | 0.038 | 0.038 | 0.014 |
| % calories from UPF | 0.047 | 0.044 | 0.011 |
| Alcohol volume | 0.023 | 0.006 | 0.005 |
| OOH frequency | -0.002 | -0.007 | 0.017 |

| **Table S2**. Bivariate associations in take-home sample | | | | | | |
| --- | --- | --- | --- | --- | --- | --- |
|  | Purchase occasions | Total calories | Calories from fruit & vegetables | Calories from HFSS | Calories from UPF | Volume of alcoholic beverages |
| Region ^a^ | t=1.91, df=2116, p=0.056 | t=-3.40, df=2109.6, p<0.001 | t=4.66, df=2023.8, p<0.001 | t=-3.74, df=2112.5, p<0.001 | t=-5.22, df=2103.3, p<0.001 | t=-6.13, df=1773.9, p<0.001 |
| Age ^b^ | rho=0.24, p<0.001 | rho=0.47, p<0.001 | rho=-0.27, p<0.001 | rho=0.43, p<0.001 | rho=0.40, p<0.001 | rho=0.18, p<0.001 |
| Sex ^a^ | t=-1.89, df=980.16, p=0.059 | t=-1.79, df=968.9, p=0.073 | t=-29.32, df=1001.3, p=0.747 | t=-1.18, df=968.1, p=0.238 | t=-1.34, df=964.3, p=0.182 | t=-1.65, df=954.1, p=0.099 |
| Children ^a^ | t=-5.57, df=1376.3, p<0.001 | t=-23.52, df=1925.1, p<0.001 | t=-16.51, df=2009.4, p<0.001 | t=-20.76, df=1974.2, p<0.001 | t=-18.85, df=1855.6, p<0.001 | t=-5.21, df=1471.9, p<0.001 |
| Household size ^b^ | rho=-0.06, p=0.010 | rho=-0.50, p<0.001 | rho=-0.38, p<0.001 | rho=-0.44, p<0.001 | rho=-0.43, p<0.001 | rho=-0.10, p<0.001 |
| Social grade ^c^ | X=20.24, df=16, p=0.210 | X=71.77, df=16, p<0.001 | X=28.67, df=16, p=0.026 | X=92.28, df=16, <0.001 | X=92.28, df=16, p<0.001 | X=39.25, df=12, p<0.001 |
| Purchase occasions ^b^ |  |  |  |  |  |  |
| Total calories ^b^ | rho=0.28, p<0.001 |  |  |  |  |  |
| Calories from fruit & vegetables ^b^ | rho=0.15, p<0.001 | rho=0.50, p<0.001 |  |  |  |  |
| Calories from HFSS ^b^ | rho=0.27, p<0.001 | rho=0.94, p<0.001 | rho=-0.36, p<0.001 |  |  |  |
| Calories from UPF ^b^ | rho=0.26, p<0.001 | rho=0.89, p<0.001 | rho=0.27, p<0.001 | rho=0.89, p<0.001 |  |  |
| Volume of alcoholic beverages ^b^ | rho=0.13, p<0.001 | rho=0.30, p<0.001 | rho=0.14, p<0.001 | rho=0.26, p<0.001 | rho=0.22, p<0.001 |  |
| All supermarket density ^b^ | rho=0.08, p<0.001 | rho=-0.07, p=0.001 | rho=0.01, p=0.743 | rho=-0.07, p=0.002 | rho=-0.09, p<0.001 | rho=-0.16, p<0.001 |
| Chain supermarket density ^b^ | rho=0.06, p=0.008 | rho=-0.02, p=0.362 | rho=0.01, p=0.694 | rho=-0.01, p=0.603 | rho=-0.02, p=0.370 | rho=-0.08, p<0.001 |
| Independent supermarket density ^b^ | rho=0.07, p=0.002 | rho=-0.08, p<0.001 | rho=0.01, p=0.517 | rho=-0.08, p<0.001 | rho=-0.11, p<0.001 | rho=-0.17, p<0.001 |
| All supermarket distance ^b^ | rho=-0.08, p<0.001 | rho=0.04, p=0.075 | rho=-0.01, p=0.754 | rho=0.03, p=0.116 | rho=0.05, p=0.014 | rho=0.10, p<0.001 |
| Chain supermarket distance ^b^ | rho=-0.06, p=0.006 | rho=0.03, p=0.169 | rho=-0.01, p=0.667 | rho=0.03, p=0.175 | rho=0.04, p=0.092 | rho=0.08, p<0.001 |
| Independent supermarket distance ^b^ | rho=-0.08, p<0.001 | rho=0.04, p=0.052 | rho=-0.02, p=0.334 | rho=0.04, p=0.071 | rho=0.07, p=0.002 | rho=0.12, p<0.001 |
| OOH outlet density ^b^ | rho=0.06, p=0.004 | rho=-0.05, p=0.020 | rho=0.03, p=0.186 | rho=-0.06, p=0.010 | rho=-0.08, p<0.001 | rho=-0.11, p<0.001 |
| Restaurant density ^b^ | rho=0.06, p=0.005 | rho=-0.06, p=0.010 | rho=0.05, p=0.014 | rho=-0.06, p=0.003 | rho=-0.10, p<0.001 | rho=-0.11, p<0.001 |
| Takeaway outlet density ^b^ | rho=0.05, p=0.030 | rho=-0.04, p=0.075 | rho=-0.01, p=0.511 | rho=-0.04, p=0.068 | rho=-0.05, p=0.012 | rho=-0.09, p<0.001 |
| OOH outlet distance ^b^ | rho=-0.05, p=0.022 | rho=0.05, p=0.025 | rho=0.01, p=0.776 | rho=0.04, p=0.061 | rho=0.06, p=0.008 | rho=0.11, p<0.001 |
| Restaurant distance ^b^ | rho=-0.05, p=0.012 | rho=0.04, p=0.049 | rho=-0.02, p=0.409 | rho=0.04, p=0.075 | rho=0.07, p=0.002 | rho=0.11, p<0.001 |
| Takeaway outlet distance ^b^ | rho=-0.05, p=0.017 | rho=0.04, p=0.047 | rho=0.01, p=0.801 | rho=0.03, p=0.088 | rho=0.05, p=0.022 | rho=0.10, p<0.001 |
| Composition of food environment ^c^ | X=16.27, df=8, p=0.039 | X=1.97, df=8, p=0.982 | X=20.41, df=8, p=0.009 | X=5.74, df=8, p=0.677 | X=10.57, df=8, p=0.227 | X=12.64, df=6, p=0.049 |

HFSS = high in fat, salt and sugar; OOH = out-of-home; UPF = ultra-processed food. Results (test statistic/effect size and estimated p-value) of bivariate analyses among the study variables. Superscripts indicate the test used.

^a^ Welch two sample t-test

^b^ Spearman rank correlation

^c^ Chi square test. Purchase measures were categorised into quantiles to reduce the number of parameters.

| **Table S3**. Bivariate associations among out-of-home sample | |
| --- | --- |
|  | OOH occasions |
| Region ^a^ | t=0.42, df=425.5, p=0.676 |
| Age ^b^ | rho=0.07, p=0.142 |
| Sex ^a^ | t=-2.33, df=179.9, p=0.021 |
| Children ^a^ | t=-0.55, df=231.8, p=0.582 |
| Household size ^b^ | rho=-0.11, p=0.020 |
| Social grade ^c^ | X=18.19, df=16, p=0.313 |
| All supermarket density ^b^ | rho=-0.07, p=0.153 |
| Chain supermarket density ^b^ | rho=-0.08, p=0.108 |
| Independent supermarket density ^b^ | rho=-0.06, p=0.179 |
| All supermarket distance ^b^ | rho=0.02, p=0.736 |
| Chain supermarket distance ^b^ | rho=0.03, p=0.584 |
| Independent supermarket distance ^b^ | rho=0.02, p=0.746 |
| OOH outlet density ^b^ | rho=-0.08, p=0.084 |
| Restaurant density ^b^ | rho=-0.07, p=0.137 |
| Takeaway outlet density ^b^ | rho=-0.09, p=0.058 |
| OOH outlet distance ^b^ | rho=0.03, p=0.565 |
| Restaurant distance ^b^ | rho=0.03, p=0.516 |
| Takeaway outlet distance ^b^ | rho<0.01, p=0.923 |
| Composition of food environment ^c^ | X=7.83, df=8, p=0.450 |

OOH = out-of-home. Results (test statistic/effect size and estimated p-value) of bivariate analyses among the study variables. Superscripts indicate the test used.

^a^ Welch two sample t-test

^b^ Spearman rank correlation

^c^ Chi square test. Purchase measures were categorised into quantiles to reduce the number of parameters

| **Table S4**. Associations between area characteristics and food environment exposure in take-home and OOH sample | | | | |
| --- | --- | --- | --- | --- |
|  | Area deprivation | | Population density | |
|  | Take-home sample | OOH sample | Take-home sample | OOH sample |
| Supermarket density | rho=-0.27, p<0.001 | rho=-0.30, p<0.001 | rho=0.72, p<0.001 | rho=0.75, p<0.001 |
| Supermarket distance | rho=0.16, p<0.001 | rho=0.16, p<0.001 | rho=-0.56, p<0.001 | rho=-0.58, p<0.001 |
| Chain supermarket density | rho=-0.17, p<0.001 | rho=-0.22, p<0.001 | rho=0.50, p<0.001 | rho=0.56, p<0.001 |
| Chain supermarket distance | rho=0.12, p<0.001 | rho=0.13, p=0.008 | rho=-0.49, p<0.001 | rho=-0.50, p<0.001 |
| Independent supermarket density | rho=-0.26, p<0.001 | rho=-0.27, p<0.001 | rho=0.73, p<0.001 | rho=0.74, p<0.001 |
| Independent supermarket distance | rho=0.21, p<0.001 | rho=0.19, p<0.001 | rho=-0.64, p<0.001 | rho=-0.65, p<0.001 |
| OOH outlet density | rho=-0.10, p<0.001 | rho=-0.13, p=0.006 | rho=0.65, p<0.001 | rho=0.69, p<0.001 |
| OOH outlet distance | rho=0.16, p<0.001 | rho=0.17, p<0.001 | rho=-0.55, p<0.001 | rho=-0.55, p<0.001 |
| Restaurant density | rho=0.02, p=0.296 | rho=-0.01, p=0.785 | rho=0.63, p<0.001 | rho=0.67, p<0.001 |
| Restaurant distance | rho=0.06, p=0.008 | rho=0.08, p=0.091 | rho=-0.62, p<0.001 | rho=-0.61, p<0.001 |
| Takeaway outlet density | rho=-0.24, p<0.001 | rho=-0.28, p<0.001 | rho=0.56, p<0.001 | rho=0.59, p<0.001 |
| Takeaway outlet distance | rho=0.18, p<0.001 | rho=0.19, p<0.001 | rho=-0.54, p<0.001 | rho=-0.54, p<0.001 |
| Food environment composition | Kruskal-Wallis chi-squared=116.9, df=2, p<0.001 | Kruskal-Wallis chi-squared=21.5, df=2, p<0.001 | Kruskal-Wallis chi-squared=311.1, df=2, p<0.001 | Kruskal-Wallis chi-squared=86.1, df=2, p<0.001 |

OOH = out-of-home. Spearman rank correlation for all associations except those concerning the food environment composition, which were tested using Kruskal-Wallis test.

| Table S5. Associations between region and food environment exposure | | |
| --- | --- | --- |
| Exposure measure | Take-home sample | Out-of-home sample |
| Density of all supermarkets | t=23.93, df=1547.2, p<0.001 | t=10.94, df=298.0, p<0.001 |
| Distance to nearest supermarket (any) | t=-14.17, df=1286.7, p<0.001 | t=-7.86, df=340.7, p<0.001 |
| Density of chain supermarkets | t=10.81, df=2115.7, p<0.001 | t=5.09, df=443.7, p<0.001 |
| Distance to nearest chain supermarket | t=-12.81, df=1268.1, p<0.001 | t=-6.63, df=312.2, p<0.001 |
| Density of independent supermarkets | t=25.36, df=1330.3, p<0.001 | t=11.46, df=255.5, p<0.001 |
| Distance to nearest independent supermarket | t=-17.79, df=1206.2, p<0.001 | t=-9.07, df=300.9, p<0.001 |
| Density of OOH outlets | t=15.75, df=1816.8, p<0.001 | t=7.40, df=435.1, p<0.001 |
| Distance to nearest OOH outlet | t=-14.07, df=1249.1, p<0.001 | t=-6.83, df=305.1, p<0.001 |
| Density of restaurants | t=16.17, df=1693.1, p<0.001 | t=7.79, df=429.0, p<0.001 |
| Distance to nearest restaurant | t=-19.19, df=1212.1, p<0.001 | t=-9.18, df=291.8, p<0.001 |
| Density of takeaway outlets | t=9.01, df=2107.9, p<0.001 | t=3.99, df=442.7, p<0.001 |
| Distance to nearest takeaway outlets | t=-13.49, df=1241.7=, p<0.001 | t=-6.56, df=299.6, p<0.001 |
| Food environment composition | X=139.38, df=2, p<0.001 | X=32.92, df=2, p<0.001 |

OOH = out-of-home.

| **Table S6**. Parameter estimates and 95% CI of interaction terms between food environment exposure and region on the effect of take-home purchase outcomes | | | | | | | | | | | | | | | | | | | | | | | | | | | | | | | |
| --- | --- | --- | --- | --- | --- | --- | --- | --- | --- | --- | --- | --- | --- | --- | --- | --- | --- | --- | --- | --- | --- | --- | --- | --- | --- | --- | --- | --- | --- | --- | --- |
|  | Frequency | | | | | Total Calories | | | | | Calories from fruit & vegetables | | | | | Calories from HFSS | | | | | Calories from UPF | | | | | Alcohol volume | | | | | |
| Exposure | IR | 95% CI | | *p* value | | IR | 95% CI | | *p* value | | IR | 95% CI | | *p* value | | IR | 95% CI | | *p* value | | IR | 95% CI | | *p* value | | IR | 95% CI | | *p* value | |  |
| Density of chain supermarkets | 1.003 | | 0.981; 1.026 | | 0.976 | 0.992 | | 0.974; 1.010 | | 0.542 | 1.004 | | 0.976; 1.033 | | 0.896 | 1.009 | | 1.000; 1.018 | | 0.144 | 1.002 | | 0.991; 1.014 | | 0.763 | 1.004 | | 0.929; 1.086 | | 0.916 |  |
| Distance to chain supermarkets | 0.953 | | 0.912; 0.995 | | 0.114 | 0.988 | | 0.954; 1.024 | | 0.542 | 0.984 | | 0.932; 1.040 | | 0.896 | 0.989 | | 0.972; 1.006 | | 0.255 | 0.988 | | 0.966; 1.010 | | 0.740 | 0.906 | | 0.779; 1.054 | | 0.537 |  |
| Density of independent supermarkets | 0.999 | | 0.984; 1.015 | | 0.976 | 1.005 | | 0.992; 1.018 | | 0.542 | 1.004 | | 0.985; 1.024 | | 0.896 | 1.005 | | 0.999; 1.011 | | 0.144 | 0.998 | | 0.990; 1.006 | | 0.763 | 0.929 | | 0.881; 0.980 | | 0.028 |  |
| Distance to independent supermarkets | 0.994 | | 0.951; 1.038 | | 0.976 | 0.979 | | 0.944; 1.015 | | 0.542 | 1.004 | | 0.949; 1.061 | | 0.896 | 0.985 | | 0.968; 1.002 | | 0.144 | 0.977 | | 0.955; 0.999 | | 0.348 | 1.008 | | 0.865; 1.175 | | 0.916 |  |
| Density of OOH outlets | 1.000 | | 0.997; 1.003 | | 0.976 | 0.999 | | 0.996; 1.002 | | 0.542 | 0.999 | | 0.995; 1.003 | | 0.896 | 1.001 | | 1.000; 1.003 | | 0.144 | 1.000 | | 0.998; 1.001 | | 0.763 | 0.995 | | 0.984; 1.007 | | 0.888 |  |
| Distance to OOH outlets | 0.934 | | 0.886; 0.984 | | 0.089 | 0.983 | | 0.942; 1.027 | | 0.542 | 0.977 | | 0.914; 1.045 | | 0.896 | 0.994 | | 0.974; 1.015 | | 0.591 | 0.989 | | 0.963; 1.016 | | 0.763 | 0.769 | | 0.640; 0.924 | | 0.028 |  |
| Food environment composition | | | | | | | | | | | | | | | | | | | | | | | | | | | | | | |  |
| More OOH outlets | 1.003 | | 0.907; 1.109 | | 0.976 | 0.944 | | 0.869; 1.025 | | 0.542 | 0.952 | | 0.838; 1.082 | | 0.896 | 1.021 | | 0.981; 1.063 | | 0.352 | 0.992 | | 0.942; 1.045 | | 0.763 | 1.023 | | 0.721; 1.453 | | 0.916 |  |
| No outlets | 0.876 | | 0.732; 1.048 | | 0.394 | 0.805 | | 0.695; 0.933 | | 0.031 | 0.943 | | 0.750; 1.184 | | 0.896 | 0.914 | | 0.851; 0.982 | | 0.113 | 0.924 | | 0.842; 1.013 | | 0.364 | 1.178 | | 0.629; 2.204 | | 0.916 |  |

95% CI = 95% confidence interval; HFSS = high in fat, salt and sugar; IR = Incidence Rate; OOH = out of home; UPF = ultra-processed foods. London is coded as the baseline region. All models are adjusted for age, sex and NRS social grade of the main shopper, number of children and adults in the household, region, area deprivation and population density, and interactions between region and NRS social grade, area deprivation, and population density. *p* values were adjusted for multiple testing using the Benjamini-Hochberg method.

| **Table S7**. Parameter estimates and 95% CI of interaction terms between food environment exposure and region on the effect of OOH purchasing | | | |
| --- | --- | --- | --- |
| Exposure | IR | 95% CI | *p* value |
| Density of all supermarkets | 0.980 | 0.940; 1.021 | 0.960 |
| Distance to any supermarket | 1.006 | 0.790; 1.282 | 0.960 |
| Density of restaurants | 0.996 | 0.978; 1.014 | 0.960 |
| Distance to restaurants | 0.925 | 0.746; 1.148 | 0.960 |
| Density of takeaway outlets | 0.982 | 0.940; 1.026 | 0.960 |
| Distance to takeaway outlets | 1.014 | 0.831; 1.236 | 0.960 |
| Composition of food environments |  |  |  |
| More OOH | 1.249 | 0.803; 1.944 | 0.960 |
| No outlets | 1.039 | 0.448; 2.412 | 0.960 |

95% CI = 95% confidence interval; OOH = out of home; IR = Incidence Rate. London is coded as the baseline region.

All models are adjusted for age, sex NRS social grade, number of children and adults in the household, region, area deprivation and population density, and interactions between region and NRS social grade, area deprivation, and population density. *p* values were adjusted for multiple testing using the Benjamini-Hochberg method.

Sensitivity Analysis concerning varying buffer sizes (0.5, 1, 2, and 5 km), aggregations of supermarket classifications, and including purchases from individuals other than the main OOH reporter per household. Unadjusted *p* values are presented.

**1. Buffer size**

| **Table S8**. Sensitivity analysis of varying buffer sizes applied to selected models | | | | | | | | | | | | | |
| --- | --- | --- | --- | --- | --- | --- | --- | --- | --- | --- | --- | --- | --- |
| Model (exposure & outcome) | 1 km buffer | | | 0.5 km buffer | | | 2 km buffer | | | 5 km buffer | | | |
|  | IR | 95% CI | *p* value | IR | 95% CI | *p* value | IR | 95% CI | *p* value | IR | 95% CI | *p* value |  |
| OOH outlet density & frequency | 1.001 | 0.999; 1.003 | 0.235 | 1.001 | 1.000; 1.001 | 0.217 | 1.002 | 1.000; 1.005 | 0.038 | 1.002 | 0.998; 1.006 | 0.292 |  |
| Independent supermarket density & total calories | 1.001 | 0.996; 1.005 | 0.760 | 1.001 | 0.999; 1.004 | 0.391 | 0.997 | 0.988; 1.006 | 0.467 | 0.991 | 0.977; 1.006 | 0.235 |  |
| Chain supermarket density & calories from fruit and vegetables | 0.999 | 0.985; 1.013 | 0.903 | 1.005 | 0.998; 1.012 | 0.202 | 0.984 | 0.960; 1.008 | 0.196 | 1.002 | 0.955; 1.050 | 0.946 |  |
| Independent supermarket density & calories from HFSS | 0.998 | 0.995; 1.000 | 0.034 | 1.000 | 0.998; 1.001 | 0.510 | 0.997 | 0.993; 1.001 | 0.168 | 0.999 | 0.992; 1.006 | 0.712 |  |
| OOH outlet density & calories from UPF | 1.000 | 0.999; 1.001 | 0.648 | 1.000 | 1.000; 1.001 | 0.454 | 1.000 | 0.999; 1.001 | 0.947 | 1.001 | 1.000; 1.003 | 0.135 |  |
| Chain supermarket density & alcohol volume | 0.965 | 0.928; 1.004 | 0.074 | 0.986 | 0.967; 1.005 | 0.141 | 1.014 | 0.947; 1.086 | 0.683 | 1.149 | 1.009; 1.309 | 0.036 |  |
| Restaurant density & OOH purchasing | 0.989 | 0.980; 0.998 | 0.020 | 0.997 | 0.992; 1.002 | 0.223 | 0.990 | 0.976; 1.004 | 0.170 | 0.992 | 0.974; 1.010 | 0.400 |  |

Effect estimates of density measures refer to a change in incidence rate in response to an increase of 1 m/km^2^. All models are adjusted for age, sex NRS social grade, number of children and adults in the household, region, area deprivation and population density, and interactions between region and NRS social grade, area deprivation, and population density. Note that *p* values have not been adjusted for multiple testing.

**2. Varying aggregations of supermarket definitions**

| **Table S9**. Sensitivity analysis of effects of varying aggregations of supermarket definitions on take-home purchase outcomes | | | | | | | | | | | | | | | | | | | | | | | | | | | | | | | |
| --- | --- | --- | --- | --- | --- | --- | --- | --- | --- | --- | --- | --- | --- | --- | --- | --- | --- | --- | --- | --- | --- | --- | --- | --- | --- | --- | --- | --- | --- | --- | --- |
|  | Adjusted Estimates | | | | | | | | | | | | | | | | | | | | | | | | | | | | | | |
|  | Frequency | | | | | Total Calories | | | | | Calories from fruit & vegetables | | | | | Calories from HFSS | | | | | Calories from UPF | | | | | Alcohol volume | | | | | |
| Exposure | IR | 95% CI | | *p* value | | IR | 95% CI | | *p* value | | IR | 95% CI | | *p* value | | IR | 95% CI | | *p* value | | IR | 95% CI | | *p* value | | IR | 95% CI | | *p* value | |  |
| A density | 1.003 | | 0.987; 1.020 | | 0.689 | 1.005 | | 0.991; 1.018 | | 0.510 | 1.000 | | 0.979; 1.021 | | 0.981 | 1.003 | | 0.996; 1.010 | | 0.381 | 1.006 | | 0.997; 1.014 | | 0.190 | 0.973 | | 0.918; 1.031 | | 0.350 |  |
| A distance | 0.994 | | 0.983; 1.005 | | 0.261 | 1.001 | | 0.993; 1.010 | | 0.747 | 1.004 | | 0.990; 1.018 | | 0.542 | 0.998 | | 0.993; 1.002 | | 0.305 | 0.994 | | 0.988; 0.999 | | 0.033 | 0.998 | | 0.960; 1.036 | | 0.907 |  |
| B density | 1.011 | | 0.993; 1.029 | | 0.246 | 1.010 | | 0.995; 1.025 | | 0.188 | 0.998 | | 0.976; 1.021 | | 0.866 | 1.005 | | 0.998; 1.012 | | 0.190 | 1.007 | | 0.998; 1.016 | | 0.147 | 0.944 | | 0.887; 1.004 | | 0.067 |  |
| B distance | 0.988 | | 0.977; 0.998 | | 0.017 | 1.002 | | 0.993; 1.010 | | 0.683 | 1.002 | | 0.989; 1.015 | | 0.762 | 0.999 | | 0.995; 1.003 | | 0.513 | 0.994 | | 0.988; 0.999 | | 0.017 | 1.002 | | 0.967; 1.038 | | 0.929 |  |
| C density | 0.998 | | 0.993; 1.004 | | 0.532 | 1.001 | | 0.996; 1. 005 | | 0.760 | 1.002 | | 0.995; 1.009 | | 0.553 | 0.998 | | 0.995; 1.000 | | 0.033 | 0.998 | | 0.995; 1.001 | | 0.114 | 0.979 | | 0.960; 0.998 | | 0.028 |  |
| C distance | 0.989 | | 0.979; 1.000 | | 0.055 | 0.998 | | 0.989; 1.007 | | 0.699 | 1.001 | | 0.988; 1.015 | | 0.840 | 1.000 | | 0.995; 1.004 | | 0.832 | 0.996 | | 0.991; 1.002 | | 0.202 | 0.996 | | 0.959; 1.034 | | 0.831 |  |
| Chains density | 1.006 | | 0.994; 1.017 | | 0.323 | 1.006 | | 0.997; 1.015 | | 0.203 | 0.999 | | 0.985; 1.013 | | 0.903 | 1.003 | | 0.999; 1.008 | | 0.159 | 1.005 | | 0.999; 1.011 | | 0.074 | 0.965 | | 0.928; 1.004 | | 0.074 |  |
| Chains distance | 0.989 | | 0.977; 1.002 | | 0.094 | 1.002 | | 0.992; 1.013 | | 0.692 | 1.004 | | 0.988; 1.020 | | 0.636 | 0.998 | | 0.993; 1.003 | | 0.452 | 0.993 | | 0.987; 1.000 | | 0.040 | 1.004 | | 0.960; 1.049 | | 0.875 |  |
| All density | 1.000 | | 0.995; 1.004 | | 0.921 | 1.001 | | 0.998; 1.005 | | 0.459 | 1.001 | | 0.996; 1.007 | | 0.673 | 0.999 | | 0.997; 1.001 | | 0.247 | 0.999 | | 0.997; 1.002 | | 0.579 | 0.981 | | 0.966; 0.996 | | 0.014 |  |
| All distance | 0.983 | | 0.968; 0.998 | | 0.027 | 0.999 | | 0.986; 1.012 | | 0.868 | 1.006 | | 0.986; 1.026 | | 0.570 | 0.997 | | 0.991; 1.004 | | 0.415 | 0.993 | | 0.985; 1.001 | | 0.087 | 0.991 | | 0.938; 1.045 | | 0.729 |  |

A = big chain supermarkets; B = small chain supermarkets & convenience symbol groups; C = independent supermarkets; Chains = A & B; all = A, B & C; 95% CI = 95% confidence interval; HFSS = high in fat, salt and sugar; IR = Incidence Rate; OOH = out of home; UPF = ultra-processed foods.

Effect estimates of density measures refer to a change in incidence rate in response to an increase of 1 m/km^2^. Effect estimates of distance measures refer to a change in incidence rate in response to an increase of 500 m. The reference category for the composition of food environments is neighbourhoods with more supermarkets.

All models are adjusted for age, sex and NRS social grade of the main shopper, number of children and adults in the household, region, area deprivation and population density, and interactions between region and NRS social grade, area deprivation, and population density. Note that *p* values have not been adjusted for multiple testing.

| **Table S10**. Sensitivity analysis of effects of varying aggregations of supermarket definitions on OOH purchasing | | | |
| --- | --- | --- | --- |
| Exposure | IR | 95% CI | *p* value |
| A density | 0.910 | 0.848; 0.977 | 0.010 |
| A distance | 1.021 | 0.974; 1.069 | 0.385 |
| B density | 0.969 | 0.899; 1.045 | 0.421 |
| B distance | 0.979 | 0.929; 1.032 | 0.433 |
| C density | 0.983 | 0.961; 1.006 | 0.147 |
| C distance | 1.023 | 0.970; 1.079 | 0.397 |
| Chains density | 0.949 | 0.906; 0.994 | 0.028 |
| Chains distance | 1.000 | 0.942; 1.060 | 0.988 |
| All density | 0.979 | 0.961; 0.998 | 0.030 |
| All distance | 1.012 | 0.931; 1.101 | 0.775 |

A = big chain supermarkets; B = small chain supermarkets & convenience symbol groups; C = independent supermarkets; Chains = A & B; all = A, B & C; 95% CI = 95% confidence interval; IR = Incidence Rate; OOH = out of home.

Effect estimates of density measures refer to a change in incidence rate in response to an increase of 1 m/km^2^. Effect estimates of distance measures refer to a change in incidence rate in response to an increase of 500 m. The reference category for the composition of food environments is neighbourhoods with more supermarkets.

All models are adjusted for age, sex NRS social grade, number of children and adults in the household, region, area deprivation and population density, and interactions between region and NRS social grade, area deprivation, and population density. Note that *p* values have not been adjusted for multiple testing.

**3. Including OOH purchases reported from someone other than the main reporter**

| **Table S11**. Sensitivity analysis of including OOH purchases not reported by the main OOH reporter | | | | |  |  |
| --- | --- | --- | --- | --- | --- | --- |
|  | Only from main reporter | | | All OOH purchases | | |
| Exposure | IR | 95% CI | *p* value | IR | 95% CI | *p* value |
| Density of all supermarkets | 0.979 | 0.961; 0.998 | 0.030 | 0.985 | 0.966; 1.004 | 0.111 |
| Distance to any supermarket | 1.012 | 0.931; 1.101 | 0.775 | 1.009 | 0.928; 1.098 | 0.834 |
| Density of restaurants | 0.989 | 0.980; 0.998 | 0.020 | 0.991 | 0.982; 1.000 | 0.044 |
| Distance to restaurants | 1.005 | 0.952; 1.060 | 0.862 | 0.999 | 0.946; 1.054 | 0.961 |
| Density of takeaway outlets | 0.976 | 0.955; 0.997 | 0.022 | 0.974 | 0.954; 0.995 | 0.015 |
| Distance to takeaway outlets | 1.004 | 0.951; 1.061 | 0.875 | 1.016 | 0.962, 1.074 | 0.558 |
| Composition of food environments |  |  |  |  |  |  |
| More OOH | 0.850 | 0.685; 1.056 | 0.141 | 0.808 | 0.650; 1.004 | 0.054 |
| No outlets | 0.861 | 0.622; 1.191 | 0.365 | 0.842 | 0.608; 1.167 | 0.303 |

95% CI = 95% confidence interval; IR = Incidence Rate; OOH = out of home. Effect estimates of density measures refer to a change in incidence rate in response to an increase of 1 m/km^2^. Effect estimates of distance measures refer to a change in incidence rate in response to an increase of 500 m. The reference category for the composition of food environments is neighbourhoods with more supermarkets.

All models are adjusted for age, sex NRS social grade, number of children and adults in the household, region, area deprivation and population density, and interactions between region and NRS social grade, area deprivation, and population density. Note that *p* values have not been adjusted for multiple testing.
